# A simple two-state protein unfolds mechanically via multiple heterogeneous pathways at single-molecule resolution

**DOI:** 10.1038/ncomms11777

**Published:** 2016-06-01

**Authors:** Jörg Schönfelder, Raul Perez-Jimenez, Victor Muñoz

**Affiliations:** 1 Department of Macromolecular Structures, National Biotechnology Center, Consejo Superior de Investigaciones Científicas, Darwin 3, Campus de Cantoblanco, 28049 Madrid, Spain; 2 Nanobiosystems Programme, IMDEA Nanosciences, Faraday 9, Ciudad Universitaria Cantoblanco, 28049 Madrid, Spain; 3 Nanobiomechanics Laboratory, CIC nanoGUNE, 20018 San Sebastián, Spain; 4 IKERBASQUE, Basque Foundation for Science, 48013 Bilbao, Spain; 5 Department of Bioengineering, School of Engineering, University of California, Merced, California 95343, USA

## Abstract

A major drive in protein folding has been to develop experimental technologies to resolve the myriads of microscopic pathways and complex mechanisms that purportedly underlie simple two-state folding behaviour. This is key for cross-validating predictions from theory and modern computer simulations. Detecting such complexity experimentally has remained elusive even using methods with improved time, structural or single-molecule resolution. Here, we investigate the mechanical unfolding of cold shock protein B (Csp), a showcase two-state folder, using single-molecule force-spectroscopy. Under controlled-moderate pulling forces, the unfolding of Csp emerges as highly heterogeneous with trajectories ranging from single sweeps to different combinations of multiple long-lived mechanical intermediates that also vary in order of appearance. Steered molecular dynamics simulations closely reproduce the experimental observations, thus matching unfolding patterns with structural events. Our results provide a direct glimpse at the nanoscale complexity underlying two-state folding, and postulate these combined methods as unique tools for dissecting the mechanical unfolding mechanisms of such proteins.

## Abstract (web summary)

Previous investigations have indicated that the model protein CspB folds in a simple two-state fashion. Here, the authors provide direct experimental evidence for that the energy landscape of two-state folding proteins is highly heterogeneous and that unfolding can occur via multiple pathways.

## Graphical abstract

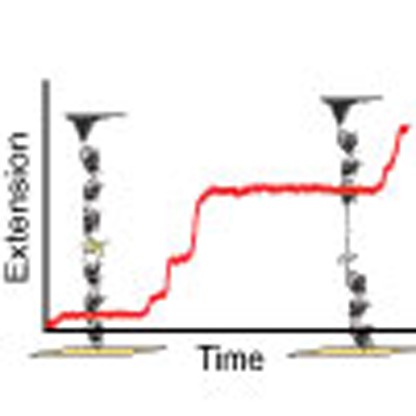

Protein folding can be portrayed as the diffusive search for the biologically functional three-dimensional structure on a corrugated folding free-energy landscape (FFEL) that has an overall funneled shape towards the native state^1^. The funnel provides the driving force, but the folding mechanisms depend critically on landscape topography^2^,^3^. Advanced atomistic computer simulations suggest that FFEL are indeed extremely complex^4^,^5^,^6^. Experiments, on the other hand, produce a simple picture in which single-domain proteins often fold via a cooperative, two-state process^7^. Additional complexity has been observed at the fringes of the two-state regime, with ultrafast folding domains adhering to the downhill folding scenario^8^,^9^,^10^ and larger proteins with structural sub-domains often populating discrete folding intermediates^11^. Such deviations are hints of the underlying complexity, and emphasize that the detailed experimental analysis of folding mechanisms requires improved methods capable of probing FFEL topography directly^12^.

Single-molecule methods have held great promise in this regard because of their potential to resolve the stochastic paths followed by individual molecules while they (un)fold^13^. However, so far single-molecule experiments seem to confirm the simplicity of two-state protein folding. For instance, single-molecule fluorescence spectroscopy, which recapitulates the isotropic (un)folding conditions of bulk experiments^14^, has revealed a binary conversion between the native and unfolded states on multiple proteins that fold in milliseconds or longer^15^. Single-molecule force spectroscopy, whether by atomic force microscopy (AFM) or by optical/magnetic tweezers, provides an alternative procedure in which individual protein molecules are mechanically pulled from their ends^16^,^17^. In these experiments the (un)folding reaction follows the direction of the applied force, which represents a well-defined reaction coordinate^18^,^19^,^20^. The vectorial character of mechanical perturbations is likely to select a handful of (un)folding routes from the myriads that are accessible to the protein in the absence of force. Such entropic reduction may favour the control and detection of transient intermediate stages^21^. Changes in pulling geometry, for example, appear to be sufficient to modify the entire (un)folding process^22^,^23^. Nevertheless, mechanical unfolding experiments in which the protein is stretched at constant velocity (that is, force-extension) have in general produced simple, one-peak unfolding processes in which the transition state is crossed after marginal stretching from the native structure^24^. These results raise the important issue of whether such simple observations reflect the true features of natural protein FFELs, which would then be smoother than what theory predicts^2^,^3^ and atomistic simulations suggest^4^,^5^,^6^, or arise from limitations related to the time resolution in AFM mechanical unfolding experiments that use the force-extension technique.

In this work we tackle this issue by investigating the mechanical unfolding pathways of the cold shock protein B from *Thermotoga maritima* (here termed Csp). Conventional bulk thermodynamics^25^, stopped-flow kinetics^26^ and mutational analysis^27^, have showcased Csp as an example of moderately fast, two-state folding. The two-state character of Csp chemical (un)folding has been confirmed in several single-molecule Förster resonance energy transfer (FRET)-spectroscopy studies^28^,^29^. Csp mechanical unfolding has also been recently investigated using the standard force-extension technique, which reported a relatively high mechanical stability and simple two-state unfolding^30^. In those experiments Csp was subjected to very rapid stretching under uncontrolled mechanical force. Here, we mechanically unfold Csp using force-clamp AFM techniques, which allow for a fine, time dependent control of the force applied to the protein^31^,^32^ and perform steered molecular dynamics (SMD) simulations^4^ to facilitate the structural interpretation of the experimental results. Our results show that under such conditions the mechanical unfolding of Csp is remarkably heterogeneous with multiple pathways and varying numbers of long-lived intermediates, thereby revealing that the folding energy landscape of a two-state folding protein does have intricate topographic texture as predicted by theory and observed in simulations.

## Results

### Mechanical extension of Csp

**Figure 1:**
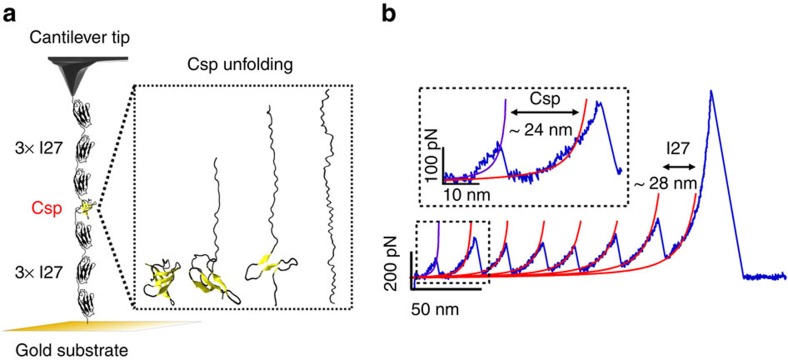
Mechanical extension of Csp. (**a**) Schematic of the (I27)_3_-Csp-(I27)_3_ polyprotein construct used in our experiments placed between a cantilever tip and the gold surface. The movement of the piezoelectric actuator allows the application of force to the polyprotein and thus investigate Csp mechanical unfolding. (**b**) A typical force-extension curve of the polyprotein sample.

To investigate the nanomechanics of Csp unfolding, we built a polyprotein consisting of one Csp domain (65 aa) flanked by molecular handles, each made of three repeats of the I27 domain of human cardiac titin (Fig. 1). The I27 domains provide a mechanical fingerprint with well-characterized contour length and unfolding force^33^. This general approach has been successful in investigating the mechanical unfolding of many other proteins^34^, as well as of Csp^30^. For direct comparison with the previous AFM study, we performed standard force-extension experiments by stretching the polyprotein at a constant speed of 400 nm s^−1^. From these experiments we determined a contour length of (24.1±0.9) nm and an unfolding force of (81.4±22.2) pN for Csp (Supplementary Fig. 1) that are consistent with previous reports^30^. Intriguingly, a small fraction of the traces (<10%) showed signs of an unfolding intermediate (Supplementary Fig. 1b). This intermediate, however, did not exhibit a defined length pattern (Supplementary Fig. 1c).

### Unveiling the complexity of Csp mechanical unfolding

**Figure 2:**
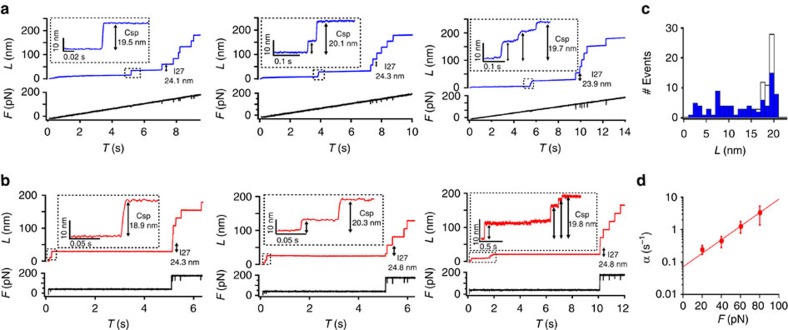
Force-ramp and force-clamp experiments on Csp. (**a**) Three examples of force-ramp traces at 20 pN s^−1^ in which the force is ramped until 300 pN to trigger the final unfolding of the six I27 domains. (**b**) Three examples of force-clamp traces at 40 pN for up to 10 s followed by a jump to 150 pN to trigger the unfolding of the six I27 domains. (**c**) Distribution of unfolding lengths of Csp intermediates obtained from force-ramp measurements (bin size 1 nm). The total unfolding length from each trace is also shown as empty bars. (**d**) Semi-log plot of the unfolding rate (*α*) versus force (error bars in s.d.). With length (*L*), force (*F*) and time (*T*).

To investigate the mechanical unfolding of Csp in further detail, we turned to AFM methods in which the force is slowly increased at a constant rate (force ramp), see Methods^31^,^32^. Force-ramp experiments afford better temporal control, and are thus more likely to reveal details that might not be apparent in AFM experiments conducted at constant pulling velocity. In contrast to force extension, Csp unfolding is clearly distinguishable in force-ramp experiments because it occurs much later than surface detachment and much earlier than unfolding of I27 repeats (Fig. 2a). Force-ramp experiments at a speed of 20 pN s^−1^ unveiled surprisingly heterogeneous unfolding patterns. In a large fraction of the trajectories Csp unfolded via a single step of ∼19 nm that is consistent with complete stretching of Csp under these conditions, that is without noticeable intermediates (for example, first trace of Fig. 2a). However, in the remaining trajectories, Csp unfolded in several steps (intermediates) (for example, second and third traces in Fig. 2a). Overall, we observed an extremely wide range of behaviours in which the number of mechanical intermediates varied from just 1 to 4 (note that number of intermediates is number of steps-1). Intermediates populated over a broad range of forces (10–110 pN, Supplementary Fig. 2b) and their extension varied from 2 nm to the full Csp extension (Fig. 2c). Analysis of all such traces indicated that the total unfolding length and force are constant at values of 19±1 nm (Fig. 2c and Supplementary Fig. 2a) and 59±22 pN (Supplementary Fig. 2b), respectively, regardless of trace complexity.

Sequential increases in ramp rate from 20 to 800 pN s^−1^ showed no changes in unfolding length (19±1 nm) (Supplementary Fig. 2a) and steady increases in unfolding force up to 83±29 pN recorded at 800 pN s^−1^ (Supplementary Fig. 2b). The detected forces are thus comparable to the unfolding forces measured by force extension (see previous paragraph). The overall unfolding heterogeneity was unaffected by the ramp rate (Supplementary Fig. 2c), indicating that the different patterns shown in Fig. 2 and Supplementary Fig. 2 are not caused by heterogeneity of the end states (native and mechanically unfolded), but rather reflect multiplicity of unfolding pathways.

### Force effects on Csp unfolding

During mechanical unfolding, the exerted force distorts the FFEL along an order parameter defined by the distance between the pulling ends, which may herd unfolding molecules through constrained manifolds of pathways. In such case it might be possible to redirect the flux through different paths by simply controlling the magnitude of the applied force. To test this idea we performed unfolding experiments at constant force using the AFM force-clamp mode^31^,^32^ (see Methods). These experiments recapitulated the force-ramp observations. Figure 2b shows examples of Csp unfolding traces recorded at a constant force of 40 pN, which include single-step unfolding trajectories (left trace in Fig. 2b) as well as a multi-step unfolding trajectories in which the number of intermediates changes from 1 to 4, but the total unfolding length remains unaltered (for example, middle and right traces in Fig. 2b). Experiments at different forces (20, 40, 60 and 80 pN) showed slight increases on total unfolding length with the magnitude of the force, as expected from the elastic properties of the unfolded chain (Supplementary Fig. 3a), but similarly wide distributions of unfolding behaviours (Supplementary Figs 4a and 5a).

Constant force experiments provide an opportunity to characterize the Csp unfolding rate as a function of force^21^. The extreme complexity that we observe in the mechanical unfolding of Csp makes it difficult to perform a detailed kinetic analysis. However, using a mean first passage time (MFPT) analysis it is possible to obtain an estimate of the average unfolding rate that is independent of the number of steps observed in individual traces. MFPT measures the shortest time leading to complete unfolding from each trajectory and thus it provides the simplest metric to compare with the rate obtained from bulk measurements, even if at a semi-quantitative level. The average rate (*α*) obtained at each force from the inverse of the MFPT (Supplementary Fig. 3b) exhibits a near log-linear trend as a function of force (Fig. 2d) that leads to an extrapolated Csp unfolding rate at zero force (*α*_0_) of 0.07 s^−1^. This unfolding rate is in relatively good agreement with that obtained from chemical denaturation in bulk^26^.

**Figure 3:**
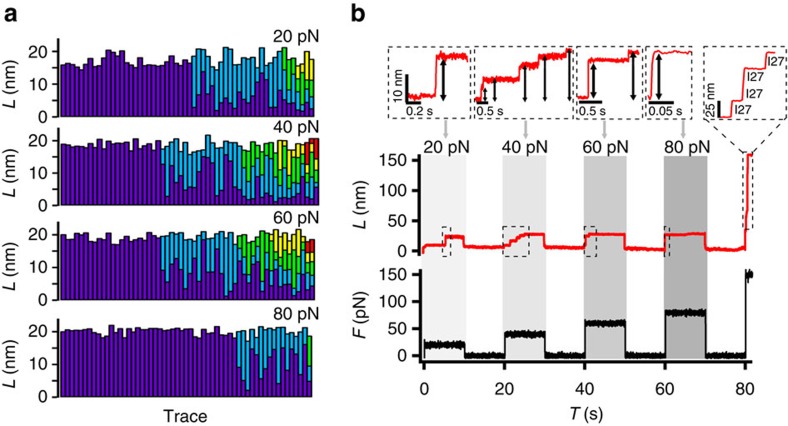
The complexity of the mechanical unfolding of Csp at the single-molecule level. (**a**) Summary of the results from single force-clamp experiments at different forces. Each vertical bar corresponds to one unfolding trace. Different colours indicate the number of intermediates observed in each trace coloured in order of appearance according to the light spectrum. The number of traces included in the analysis is 56 at 20 and 60pN, and 57 at 40 and 80 pN. (**b**) Unfolding and refolding cycles at various forces on an individual Csp molecule. The trace shows an example in which the molecule was subjected to four 10 s cycles of unfolding at various forces (20, 40, 60, 80 pN; marked by the grey swaths) intercalated by 10 s force-quenched intervals. A final jump to 150 pN is applied to trigger unfolding of the six I27 repeats. With length (*L*), force (*F*) and time (*T*).

Whereas the individual unfolding patterns for Csp are independent of the exerted force, further inspection revealed some force modulation consisting in an increase in unfolding heterogeneity (that is, average number of intermediates) as the force hikes from 20 to 40 pN, followed by a plateau between 40 and 60 pN, and a final decrease at even higher forces (Fig. 3a). These results suggest that it is indeed possible to manipulate the flux through different mechanical unfolding pathways by controlling the pulling force. To confirm that this effect is not due to sample or experimental heterogeneity, we changed the force on individual molecules in steps by performing a sequence of unfolding-refolding cycles (that is, refolding occurs during the intervals at which the force is quenched). Figure 3b shows a representative example in which Csp unfolds and refolds back during each of the four pulling-quench cycles (20, 40, 60 and 80 pN). The trace also shows the unfolding of the I27 repeats, demonstrating that the polyprotein remains attached to the cantilever for the entire experiment. This trace exemplifies the force modulation effect by showing a single Csp molecule that stochastically unfolds in a single step at 20 and 80 pN and via multiple steps at intermediate forces (Fig. 3b).

### Analysis of Csp unfolding pathways

The length distributions derived from the unfolding histograms of Csp at the various forces are all extremely broad (Supplementary Figs 4a and 5a), even though the individual traces show sharp, well delineated intermediates (Figs 2 and 3). To gain further insight we performed a cluster analysis, which showed that the data at each force could be optimally classified into six different clusters (that is, mechanical unfolding events) (see Methods). Cluster properties looked very similar for all forces with the exception of the one corresponding to the longest length (fully extended Csp), which increased at higher force likely reflecting the mechanical adaptation of the flexible unfolded state^35^. The other five clusters corresponded to unfolding intermediates with lengths that did not change significantly within the force range used in our experiments.

**Figure 4:**
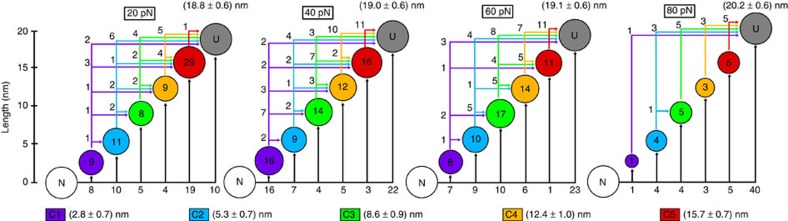
Csp unfolding intermediates and kinetic connections. Cluster analysis permits to group and classify the complex mechanical unfolding patterns of Csp in terms of a series of intermediates and the kinetic transitions between them. Average length and s.d. for each identified intermediate are listed at the bottom of the figure. The corresponding length and s.d. for the unfolded state *U* was estimated by calculating the average of the distribution of clusters C6–C8 for each individual force and is shown in each panel above the circle corresponding to *U*. The diameter of each circle (shown in the appropriate colour) is related to the number of times that intermediate was found at each force (to facilitate plotting all data on the same scale, the diameter of the circle was defined by a threshold value assigned to a single occurrence plus a value proportional to the logarithm of the total number of occurrences). The actual number of occurrences for each intermediate as a function of force is included inside the circle. Kinetic transitions from the native state to intermediates are shown as black arrows, whereas transitions from one intermediate to another one are shown as arrows in the colour of the intermediate of origin. Numbers next to each arrow indicate the number of such transitions observed.

We thus combined the data for all forces and performed a global cluster analysis to maximize the sampling of unfolding intermediates. The combined data was classified in eight clusters (C1–C8, see legend in Fig. 4). C6–C8 correspond to the extended state (U) showing different extension at low (20 pN), intermediate (40–60 pN) and high (80 pN) force, respectively. Unfolding intermediates (clusters C1–C5) were significantly populated at most forces (Fig. 4) and correspond to stepwise increases of ∼3 nm. Reconstruction of the length-distribution histograms from clustered data indicated that the identified intermediates are both well-defined (Supplementary Fig. 4b) and common for all forces (Supplementary Fig. 5b). The five distinct intermediates become more apparent when the step length data at each force is shown as a cumulative histogram (Supplementary Fig. 6).

**Figure 5:**
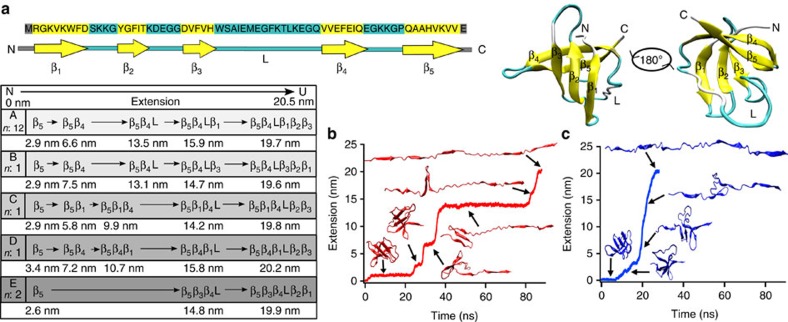
Atomistic simulations of Csp mechanical unfolding. (**a**) Sequence, topology and three-dimensional structure (PDB: 1G6P) of Csp. Residues forming the β-strands are highlighted in yellow and residues forming loops are highlighted in cyan. The overview shows the results from the pathway analysis for the 17 unfolding trajectories. (with *n* number of trajectory) (**b**) Example of a simulated trajectory representing pathway A, where Csp starts unfolding from strand 5, followed by strand 4, the loop, strand 1 and then strands 2 and 3 together. (**c**) Example of a trajectory representing pathway C, where Csp starts unfolding from strand 5, followed by strand 1 and 4, the loop and then strands 2 and 3 jointly.

Interestingly, the number of intermediates and their stepwise difference in length (∼3 nm increases) are consistent with the results to be expected for the unraveling of individual secondary structure elements in Csp (five β-strands and an ordered loop, see Fig. 5a). Experimentally we could observe simultaneously (in a single trace) up to four of the five unfolding intermediates that the analysis identifies (Supplementary Fig. 7). For the most part, individual unfolding traces show a variable number of intermediates that seem to correspond to different combinations of the six Csp secondary structure elements unfolding in groups (from just one to all of them; see Fig. 4). However, intermediates corresponding to the unfolding of one or two elements (C1 and C2) are observed somewhat more frequently than combinations that include unfolding of more (but not all) elements (C3–C5).

These results raise some important questions. One such question concerns the kinetic mechanism behind the complex mechanical unfolding patterns of Csp. The AFM data does not distinguish between secondary structure elements because they all have similar lengths. However, the kinetic connectivity among states that emerges from cluster analysis does shed some light. We see direct paths from the native state to all the intermediates (black arrows in Fig. 4) and all the possible connections among intermediates (colour arrows in Fig. 4). In fact, the number of possible connections for any given intermediate depends only on how structured it is (or how much protein chain is left to unravel). That is, each intermediate is reached from all intermediates that are more structured and feeds all intermediates that are more unfolded (Fig. 4). At the same time, the order of appearance of intermediates is not sequential, and the number of detected intermediates varies widely from trace to trace (Figs 2 and 3). Moreover, whereas for many traces all the intermediates are too transient to be detected, others show very long-lived (several seconds) intermediates (Fig. 2). Using straightforward stochastic kinetic arguments (see Methods), we can conclude that the unfolding process of Csp occurs via multiple independent pathways rather than through a single sequential pathway.

Another issue refers to the source of the force modulation of Csp unfolding. The data at different forces only differ in the probabilities of populating intermediates, which appear to become maximal between 40 and 60 pN (Figs 3 and 4). All the intermediates are more extended than the global unfolding transition state estimated from force-extension experiments (∼0.2 nm away from the native state^30^), indicating that they occur later in the unfolding pathway. In other words, the Csp intermediates we observe are kinetic traps, which suggest that the ruggedness of the FFEL increases with the pulling force up to around 50 pN, and then decays at higher forces. It is reasonable to expect that mechanically constrained folding landscapes result in enhanced steric hindrance that could increase the local barriers connecting unfolding intermediates. The question is why is such phenomenon maximal at intermediate forces? One factor to consider is that we may not be resolving all the heterogeneity present at the highest force because at that level the unfolding rate is already close to the instrumental time resolution. The fact that Csp unfolds mechanically by multiple pathways offers an alternate or complementary explanation in which the transition from low to high force could result in progressive reductions in the number of productive unfolding pathways. A relatively smooth landscape at low force such as that expected for a two-state folder would permit multiple inter-communicating microscopic pathways resulting in simple ‘time averaged' kinetics. As the productive pathways become fewer and more disconnected the unfolding kinetics should become more noticeably heterogeneous, but at some point only a single pathway will be left, thus reverting to simpler overall kinetics. Unfortunately, we cannot further investigate this possibility given the current resolution of the technique.

### Mechanistic insights from atomistic simulations

As aid for interpreting the AFM experiments, we performed SMD simulations of Csp unfolding. Here, the idea was determining whether atomistic simulations could reproduce our experimental observations, and thus provide structural and mechanistic insights about the process. We were particularly interested in mapping the time evolution of structural elements (five antiparallel β-strands and an ordered loop that connects strands 3 and 4; Fig. 5a) during unfolding. For that purpose we carried out multiple unfolding simulations using a SMD protocol at constant force (200 pN) in explicit solvent, and pulling from either the C or the N terminus (total of 17 trajectories). The main results are summarized in Fig. 5, whereas all the individual trajectories are shown in Supplementary Fig. 8. In relative terms (unfolding takes place many orders of magnitude faster in SMD simulations than in experiments), the SMD trajectories recapitulated all of the experimental observations.

Some trajectories showed very distinct, long-lived unfolding intermediates (Fig. 5b), whereas others closely resembled the experimental unfolding traces with unfolding in one step (considering the nearly infinite time resolution of MD simulations) (Fig. 5c and Supplementary Fig. 8). As in experiments, detected intermediates varied widely in length, in numbers (from 1 to 4, see Fig. 5c, b, respectively), in their relative order, and also in their dwell times relative to the global unfolding time. Statistical analysis of the simulations permits to identify five distinct intermediates (2.9±0.4 nm, 6.7±0.5 nm, 10.3±0.6 nm, 13.6±0.7 nm, 15.9±0.5 nm) in addition to the fully unfolded state (19.7±0.4 nm) (see Supplementary Fig. 8). Thus, the species observed in simulations are remarkably consistent with the experimentally determined ones (see Fig. 4). The simulated trajectories also appear as stochastically distributed as the experimental ones. Structural analysis of the simulations indicates that the shortest intermediates correspond to the unfolding of individual secondary structure elements, which are all similar in length, whereas longer intermediates result from the simultaneous unfolding of several structural elements. Therefore, the simulations directly confirmed our structural interpretation for the small and medium intermediate lengths observed experimentally (clusters C1–C5 in Fig. 4).

Interestingly, despite the limited number of trajectories, the simulations show multiple Csp unfolding pathways (see overview in Fig. 5). One particular pathway (pathway A), represented by the example in Fig. 5b, is highly predominant (found in 12 out of the 17). However, the remaining trajectories revealed a total of four additional unfolding pathways (B to E in Fig. 5). In general, all pathways start with peeling off the last (fifth) strand and differ in the order at which the other elements unfold, highlighting that Csp unfolding is indeed mechanistically heterogeneous. In the simulations, some unfolding pathways result on trajectories with long-lived intermediates, such as Fig. 5b, in which a partially unfolded intermediate containing strands 1–3 remains stably formed for over 50% of the 80 nanosecond trajectory. For other pathways, the secondary structure elements still unfold following a particular order, but they do so with much shorter time intervals between them, like the example of pathway C shown in Fig. 5c. Examples for pathways B, D and E are provided in Supplementary Fig. 8. In summary, SMD simulations buttress our experimental interpretation that the differences between unfolding trajectories occurring in a single step or through multiple intermediates reflect unfolding via different pathways.

## Discussion

The mechanical unfolding of the two-state folder Csp appears as highly heterogeneous when investigated at the single-molecule level using force-clamp AFM. The heterogeneity is manifested as a manifold of behaviours that ranges from unfolding in one step to events populating multiple mechanical intermediates, some of which remain formed for seconds during the unfolding experiment. Moreover, the degree of Csp unfolding heterogeneity depends on the pulling force (at fixed geometry), reaching apparently maximal values at ∼50 pN (Fig. 4). The heterogeneity is such that it is impossible to explain with a simple sequential pathway model. Cluster analysis of the AFM data combined with SMD simulations point instead to an unfolding process via multiple independent pathways. Under such scenario the pulling force seems to induce a redistribution of the flux among the multiple unfolding pathways of Csp in addition to tilting the landscape towards the unfolded state. This interpretation is in fact consistent with results from molecular simulations in protein G, which found shifts in the mechanical unfolding mechanism as a function of the pulling force^36^. Previous simulations have also reported heterogeneous mechanical unfolding mechanisms that change depending on the direction of the applied force^37^,^38^. From an experimental standpoint, a recently published study impinges on similar issues^39^. In that case the authors applied a multi-pronged approach that combines mechanical and chemical unfolding with mutations to infer that src SH3 unfolds via multiple unfolding pathways^39^. The similarity in conclusions obtained on diverse proteins with various approaches suggests that mechanical (un)folding through multiple pathways might be in fact a general occurrence for two-state folding proteins.

However, here we are able to go a step beyond previous studies by directly resolving such kinetic heterogeneity in single-molecule experiments. This result is remarkable because protein folding experiments almost invariably produce simple observations. For instance, in the recent src SH3 study the authors apply elegant inference to interpret changes in global unfolding patterns, but they do not resolve heterogeneous individual trajectories^39^. Existing single-molecule reports of complex behaviour are typically associated to conformational processes that depart from two-state folding even in bulk such as the single-molecule mechanical characterization of the three-state folding T4 lysozyme^40^, the molten-globule-like unfolding of a membrane-associated protein^41^, and the mechanical expansion of unfolded polyubiquitin chains that were previously collapsed by a force-quench pulse^19^. Similarly, the thermal unfolding of one-state downhill^42^ and the ultrafast folder gpW^43^,^44^ are very complex when investigated at atomic resolution because fast folding proteins have marginally cooperative unfolding processes^45^. But, Csp folds way too slowly to be in that regime^45^. Moreover, Csp robustly shows simple two-state-like (un)folding when studied with single-molecule resolution using both fluorescence detection^28^,^29^ and force-extension AFM^30^. It is only with the application of moderate, finely controlled, pulling forces in single-molecule mechanical unfolding experiments that the heterogeneity in Csp unfolding becomes apparent and comparable to that seen in atomistic computer simulations. Incidentally, our results confirm previous conclusions from theoretical analyses of kinetic^45^ and calorimetric^46^ data as well as coarse-grained folding simulations of the Csp homologue from *B. subtilis*^47^, which hinted at complexity lying under the two-state character of Csp folding. Therefore, force-clamp AFM in combination with SMD emerges as a powerful tool for probing at high resolution the topographic features of the free energy landscapes of two-state folding proteins and how these proteins respond to force perturbation.

## Methods

### Cloning and protein expression

The chimeric polyprotein construct (I27)_3_-Csp-(I27)_3_ was produced containing the DNA sequence of Csp (synthesized by Top Gene Technologies, Canada) flanked by three Titin-I27 domains on each side using standard DNA manipulation protocols to build the construct inside the pRSET A vector.

Each DNA manipulation step needed to add a protein domain consecutively into the plasmid vector was performed in sequence and confirmed by DNA sequencing (Parque Científico, Madrid). C41 strand competent cells *E*. *coli* were used for protein expression as they are specialized in expressing toxic proteins (Novagen). A gentle cell lysis protocol was used to avoid damage to the expressed polyproteins^48^. The sample was then purified by HPLC (Agilent, Santa Clara, CA) in two steps: first using a nickel-affinity HisTrap column (Ge Healthcare) and second using a size exclusion Superdex 200 column (GE Healthcare). Finally, the buffer was changed to the final buffer employed for the measurements (1 × PBS pH 7.4) using ultrafiltration Amicon 3k filters (Milipore). The final protein concentration was estimated to be around 1 mg ml^−1^ using a Nanodrop (Thermo Scientific). Then the samples were snap frozen in liquid nitrogen and stored at −80 °C.

### Single-molecule force spectroscopy

All single-molecule force spectroscopy constant force and force-ramp experiments were performed on a force-clamp AFM from Luigs Neumann^31^,^32^, whereas the force-extension measurements (see Supplementary Fig. 1) were performed with a MultiMode AFM (Bruker) equipped with a PicoForce module and a Nanocope IIIa controller. MLCT cantilevers from Bruker were used with a spring constant 30–40 pN nm^−1^ for force-extension measurements and with a spring constant 15–20 pN nm^−1^ for constant force and force-ramp measurements. The spring constant was measured before each experiment using the equipartition theorem as built in the analysis software. Data was recorded at 1 kHz for the constant velocity and between 0.5 and 4 kHz for the constant force and force-ramp measurements. During force-ramp experiments the force was ramped at the desired rate (20–800 pN s^−1^) until reaching 300 pN to ensure complete unfolding of both the sole CSP and the six mechanically sturdier Titin-I27 domains. In force-clamp experiments the Csp construct was subjected to constant forces ranging from 20 to 80 pN during 5 to 20 s followed by a hike in force (around 150 pN) to trigger the mechanical extension of the I27 mechanical fingerprint. For the force-clamp experiments with multiple cycles of unfolding-refolding, we used an experimental sequence of 10 s pulses of increasing force (20, 40, 60 and 80 pN) intercalated by periods of 10 s during which the pulling force was fully quenched. The experiment was finalized with a jump to 150 pN to trigger unfolding of the six I27 repeats.

### Experimental conditions

All AFM experiments were carried out at room-temperature (∼24 °C) in 1 × PBS buffer at pH 7.4. Typically 40 μl of the protein sample (conc. ∼μM) was left around 20 m for adsorption on a fresh gold coated surface (Arrandee). The sample was rinsed after the adsorption time with 1 × PBS buffer to remove unbounded protein sample from the gold surface just before starting the measurements.

### Data analysis

All AFM data was screened and analyzed in Igor Pro (Wavemetrics) using the built in data analysis procedure file. AFM force-extension data from the MultiMode AFM was imported into Igor Pro for further analysis. The force-extension traces were fitted to the worm-like-chain model^49^. In this model, the force *F* is given by


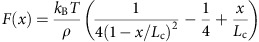


with persistence length *ρ*, contour length *L*_c_, *k*_B_ the Boltzmann constant and *T* the temperature. During the analysis the used persistence length *ρ* was between around 0.4 nm.

### Cluster analysis

We performed cluster analysis of the data using the k-means algorithm^50^, using as input data the measured lengths from all the force-clamp traces at all four forces. Particularly, we employed the k-means version implemented in Matlab (k-means++) and the Euclidean distance to define the differences in length extension (size of unfolding intermediate) between data points. To determine the number of effective clusters that best account for the heterogeneity present in the data, we performed a series of k-means runs with varying number of predefined clusters (*k*) between 2 and 10 and implemented with 20,000 replicas to guarantee convergence. We then performed a silhouette analysis of the cluster solutions for each run to identify the optimal clustering solution. The silhouette analysis calculates the average dissimilarity (*a*(*i*)) between each data point *i* and all other data within the same cluster *k* (the lower *a*(*i*) the better is the assignment) and the average dissimilarity between *i* and the closest different cluster, or neighbouring cluster (*b*(*i*)). The silhouette of data point *i* is then defined simply as: 
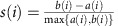
.

Silhouette values range between 1 and −1 for the best and worst cluster adscription of a given data point, respectively. To determine the optimal number of clusters we simply chose the k-means solution that resulted in the highest average silhouette for the entire dataset and minimal number of negative silhouette values for individual data points. Finally, we inspected the quality of the clustering assignments by reconstructing the length distribution cumulative histograms for each force from the average results from each cluster assuming their data points are normally distributed (Supplementary Fig. 6).

### Single sequential pathway versus multiple unfolding pathways

The results from the cluster analysis of the data at all forces indicated that the kinetic connections between intermediates are stochastically organized so that the number of possible connections coming from or going to any given intermediate depend solely on how structured is the intermediate (or how much protein chain is left to unravel) (see colour arrows in Fig. 4). In other words, we observe all connections that satisfy the relation: *L*(*C*_origin_*)+L*(*C*_destiny_)*≤L*(Csp). Accordingly, the native state extends connections to all other states; there are connections from C1 to all other intermediates plus the unfolded state; but C5 is only connected to the fully unfolded state. By the same token, there are many traces that do not show any intermediate (single steps), which indicate that in those unfolding trajectories the dwell times for all the intermediates are too short to be detected. Finally, the number of intermediates and the order of their appearance vary from trace to trace. Using simple stochastic kinetic arguments we can conclude that all of these results together rule out a single sequential unfolding pathway. The reason is that under a sequential pathway it is statistically impossible to observe both trajectories with complete unfolding in one step and others with three or four intermediates accumulating for relatively long times. In stochastic kinetic terms, the dwell times for a species that disappears via a single kinetic pathway follow an exponential distribution. Therefore, frequent observation of one-step unfolding in a process that occurs by a sequential pathway with multiple intermediates implies that the average dwell times for all those intermediates are shorter than the experimental time resolution. Because single-step trajectories are indeed very frequent for Csp, detection of one intermediate would require that its particular dwell time happened to be long enough to be observed, which implies its value must be at the tail of the exponential distribution (that is, a statistically rare event, for example, *P*<0.05). By the same argument, the probability of observing more intermediates quickly becomes infinitesimal (for example, *P*<(0.05)^2^, (0.05)^3^ and (0.05)^4^ for 2, 3 and 4 more intermediates, respectively). Furthermore, the very long-lived intermediates observed in some Csp traces (see Fig. 2) would be even much more unlikely because they require far more extreme values within the exponential distribution of dwell times. In contrast, multiple unfolding pathways can produce arbitrarily broad (non-exponential) distributions of dwell times because each pathway is characterized by a different set of kinetic transitions.

### SMD simulations

SMD simulations at a constant force of 200 pN were carried out on the Csp pdb file 1G6P. The protein structure was solvated into a waterbox (TIP3W water molecules) with the dimensions of [300/72/72] Å with a minimum distance of (20/20/20) Å from the box edge. Before starting the SMD simulation at constant force, the protein/water system was minimized using steepest descent for 2,000 timesteps and thereafter equilibrated for 1 ns using NAMD^51^ and the CHARMM22 forcefield^52^. All 17 simulations were then carried out following this single minimization and equilibration MD simulation procedure. For 13 of the SMD simulations the *C*_α_ atom of the N termini was hold fixed and 200 pN were applied on the *C*_α_ atom of the C termini of the Csp protein. For 4 SMD simulations the *C*_α_ atom of the C termini was hold fixed and 200 pN were applied on the *C*_α_ atom of the N termini of the Csp protein. SMD simulation trajectories were visualized and analyzed using the VMD software^53^.

### Data availability

The data that support the findings of this study are available from the corresponding authors upon request.

## Additional information

**How to cite this article**: Schönfelder, J. *et al*. A simple two-state protein unfolds mechanically via multiple heterogeneous pathways at single-molecule resolution. *Nat. Commun.* 7:11777 doi: 10.1038/ncomms11777 (2016).

## Supplementary Material

Supplementary Information Supplementary Figures 1 - 8
