# Supplementary material for: A simple two-state protein unfolds mechanically via multiple heterogeneous pathways at single-molecule resolution
Source: Nat Commun. 2016 Jun 1;7:11777. doi: 10.1038/ncomms11777 (PMC4895439; doi:10.1038/ncomms11777)
Supplement: Supplementary Information — Supplementary Figures 1 - 8 [file ncomms11777-s1.pdf]

Supplementary Information

A Simple Two-State Protein Unfolds Mechanically via Multiple Heterogeneous Pathways at Single-Molecule Resolution

Jörg Schönfelder, Raul Perez-Jimenez & Victor Muñoz

Supplementary Figures

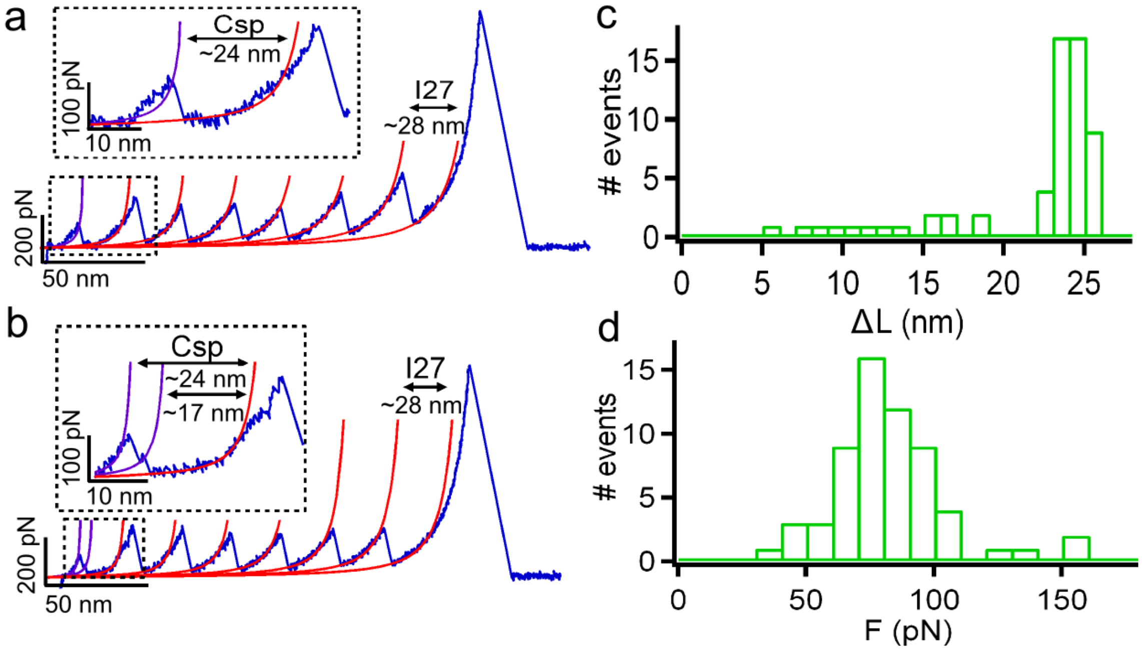

Supplementary Figure 1. Force extension AFM experiments of Csp at a pulling speed of 400 nm s^{-1} (a) Typical force extension trace showing one unfolding peak for Csp. (b) Example of force extension trace showing an intermediate during Csp unfolding (9% of a total of 56). (c) Contour length distribution (ΔL) showing a well-defined peak at 24 ± 1 nm plus a very broad distribution of smaller step lengths obtained from the minor number of traces showing and intermediate. (d) Unfolding force distribution (10 pN bin size) with average value of 81 ± 22 pN.

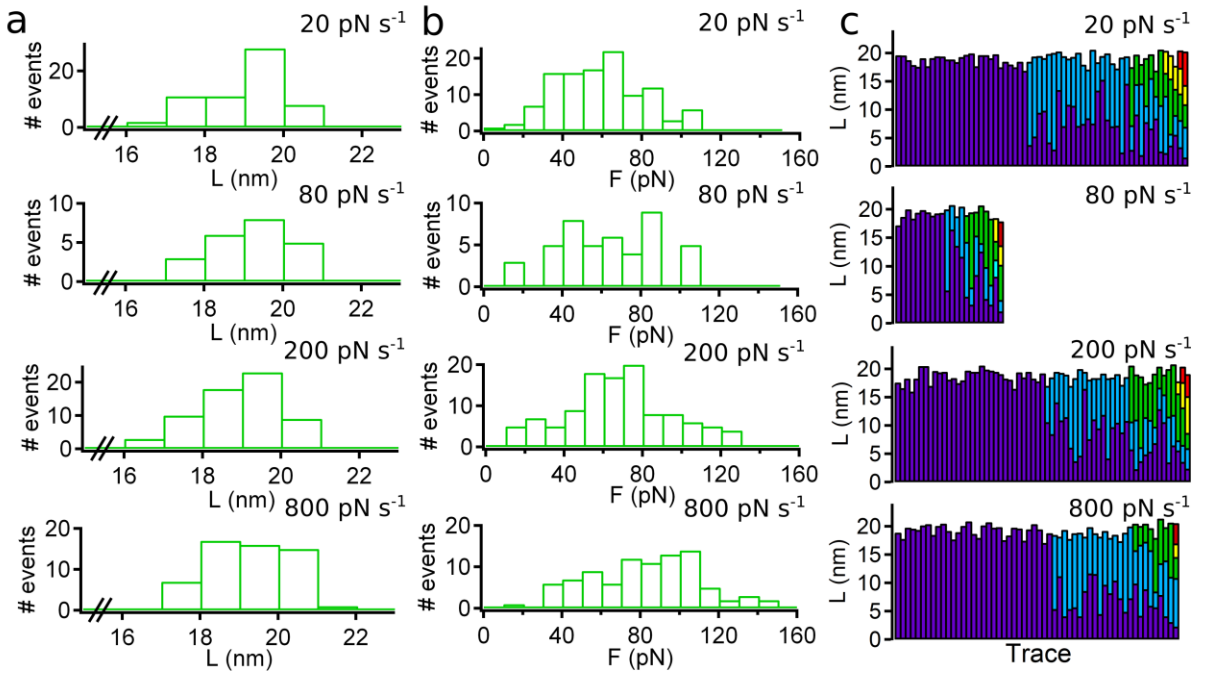

Supplementary Figure 2. Force ramp AFM measurements of Csp. Each row shows the results obtained at a given ramp rate starting from 20 pN s^{-1} (top) to 800 pN s^{-1} (bottom). (a) Total length distribution for Csp. (b) Unfolding force distribution for Csp obtained from traces that show one-step unfolding. The average unfolding force increases from 59 ± 22 pN at 20 pN s^{-1} to 62 ± 25 pN at 80 pN s^{-1} to 68 ± 27 pN at 200 pN s^{-1} to 83 ± 29 pN at 800 pN s^{-1}. (c) Graph showing the number of intermediates and their length for all the force ramp traces recorded for Csp at different ramp rates. The different colors represent the number of intermediates and their order of appearance in the unfolding pathway colored according to the light spectrum (first purple and last red). The number of traces included in the analysis is 60 at 20 pN s^{-1}, 22 at 80 pN s^{-1}, 63 at 200 pN s^{-1}, and 56 at 800 pN s^{-1}. The probability to unfold on a single step is 50 ± 5 % for the composite data at all forces.

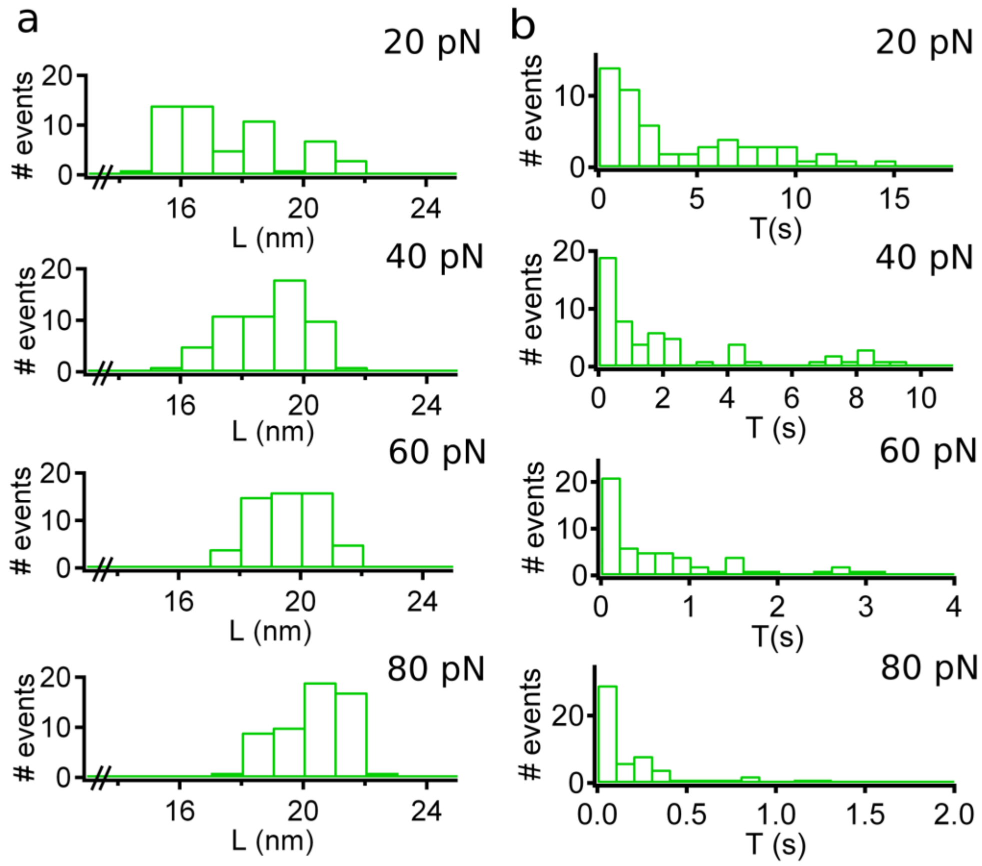

Supplementary Figure 3. Total length extension and time histograms from force-clamp experiments performed at different forces. Each row corresponds to a given force starting from 20 pN (top) to 80 pN (bottom). (Column a) Distribution of the total measured length (the sum of all steps) for Csp. The average of total unfolding length increases from 18 ± 2 nm at 20 pN to 19 ± 1nm at 40 pN to 20 ± 1 nm at 60 pN and to 20 ± 1 nm 80 pN. (Column b) Histograms of first passage times. The first passage time is defined at the earliest time at which Csp becomes completely unfolded for any given trace.

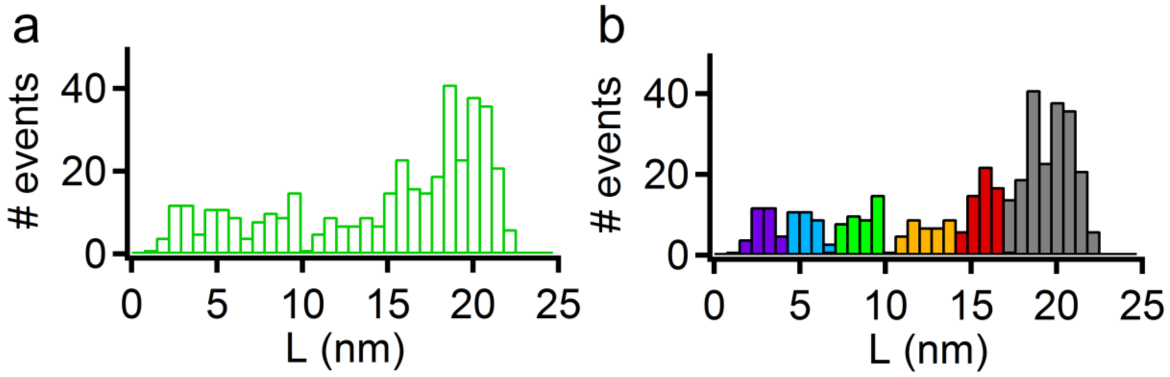

Supplementary Figure 4. Overall distribution of lengths obtained from all the steps observed in Csp force-clamp experiments at the 4 forces (20, 40, 60 and 80 pN). (a) Distribution of all measured intermediate lengths for Csp at all forces. (b) Intermediate step length distribution as in (a) but colored according to cluster ascription using the same color scheme used in main figure 4. Clusters C6 to C8 are clubbed together and colored in gray.

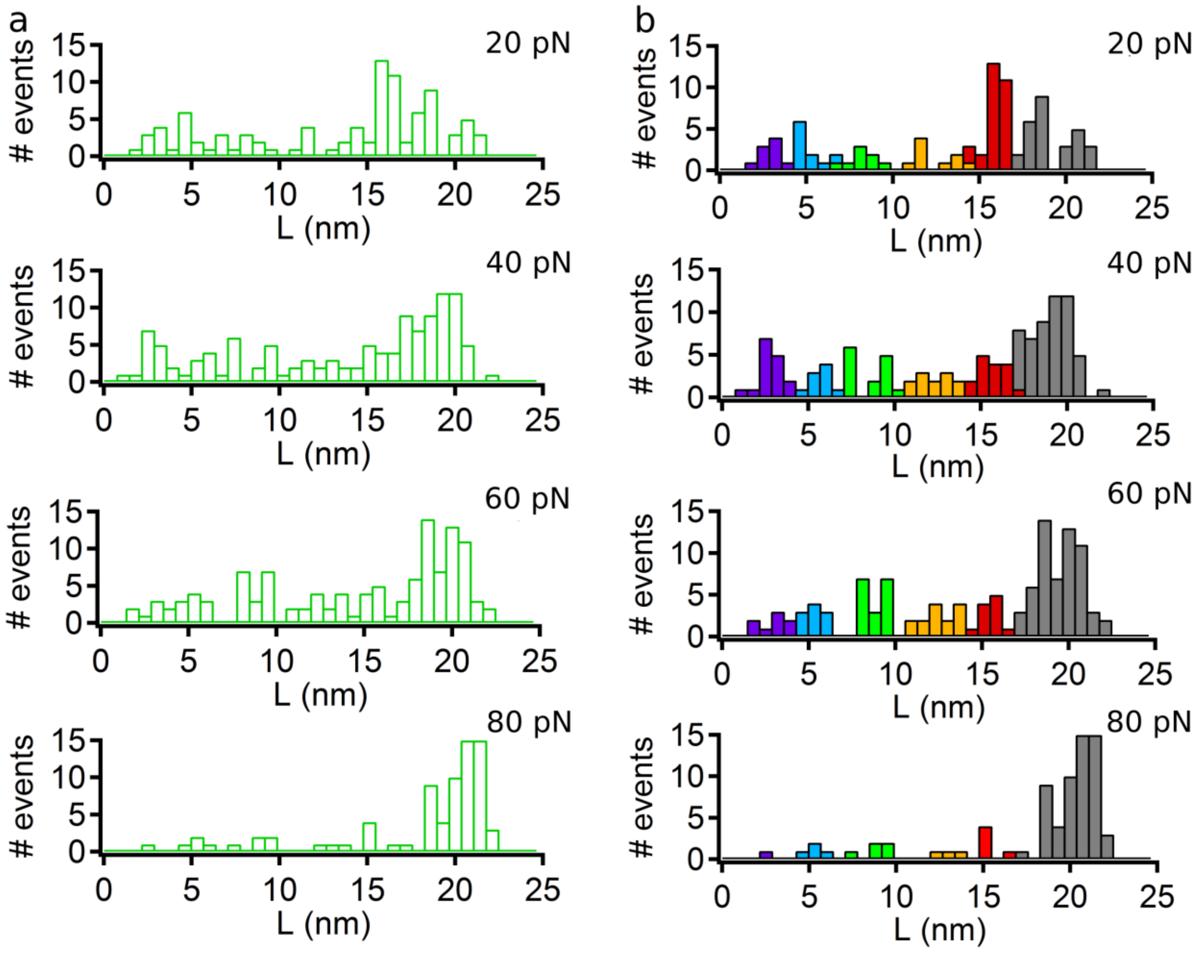

Supplementary Figure 5. Length extension histograms from force-clamp experiments performed at each force. Each row corresponds to data at a given force from 20 pN (top) to 80 pN (bottom). (Column a) Distribution of all measured intermediate step lengths for Csp. (Column b) Intermediate step length distribution as in Column a, but colored according to cluster ascription using the same color used in main figure 4. Fully unfolded events (clusters C6 to C8) are colored in gray.

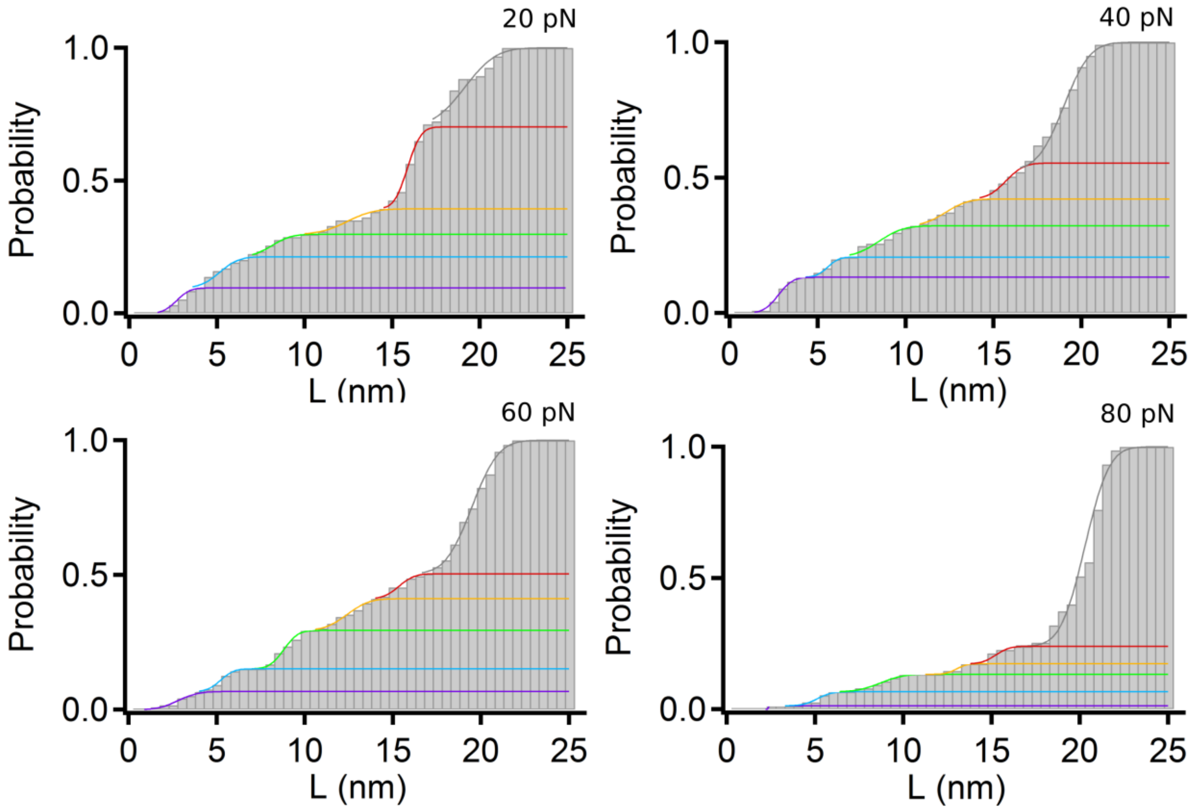

Supplementary Figure 6. Cumulative histograms showing the distribution of step lengths observed during the mechanical unfolding of Csp at constant force of 20, 40, 60 and 80 pN. Cumulative histograms facilitate the identification of stepwise intermediates in the distribution, which emerge as local plateaus. The panels also include staggered cumulative curves for each intermediate at each force (clusters C1 to C5 color coded as in main Fig. 4). The curves exemplifying the intermediates are cumulative Gaussians calculated from the cluster analysis using the number of elements, the mean and the standard deviation obtained from the elements of each cluster found at each force. The values for U (shown in gray) correspond to the combination of clusters C6 to C8.

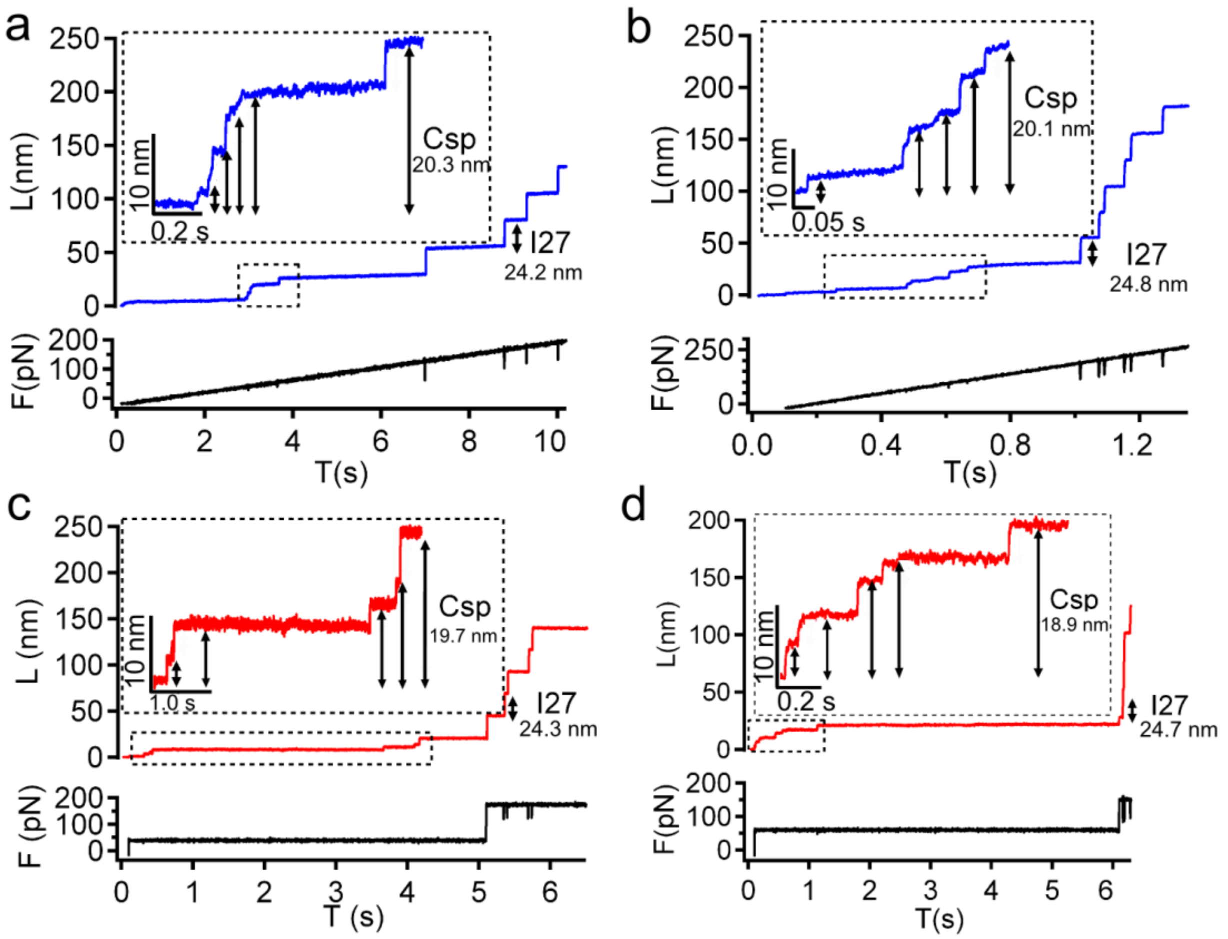

Supplementary Figure 7. Examples of Csp unfolding traces showing five steps and thus four intermediates under both force ramp (blue) and constant force (red) experiments. (a) Example of force ramp-trace recorded at 20 pN s^{-1}; (b) Example of force ramp-trace recorded at 200 pN s^{-1}; (c) Example of constant-force trace at 40 pN; (d) Example of constant-force trace at 60 pN.

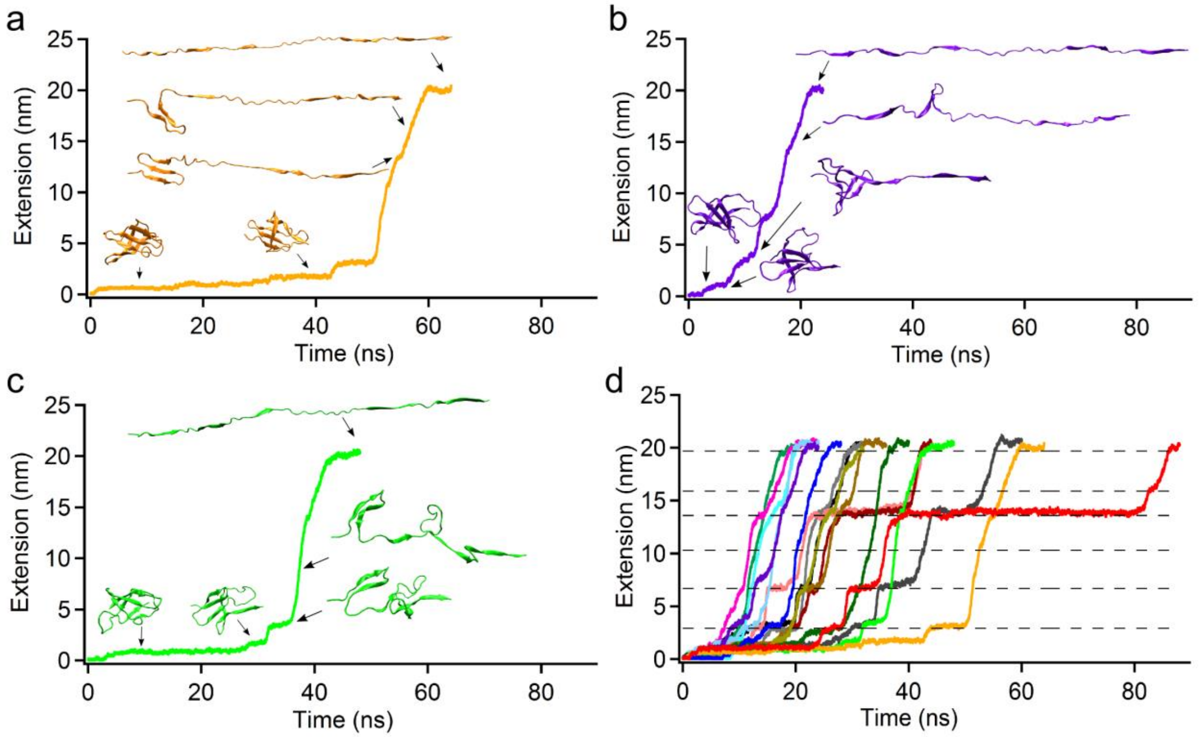

Supplementary Figure 8. Steered Molecular Dynamics trajectories of Csp performed at a constant force of 200 pN. (a) Example of a simulated trajectory showing pathway B, where Csp starts unfolding from strand 5, followed by strand 4, the loop, strand 3, and then strands 1 and 3 together. (b) Example of trajectory representing pathway D, where Csp starts unfolding from strand 5, followed by strand 4 and 1, the loop, and then strands 2 and 3 jointly. (c) Example of trajectory representing pathway E, where Csp starts unfolding from strand 5, followed by separation of strands (1+2) from the (loop+strand 3+4), unfolding of strand 3, strand 4, the loop, and finally strands 1 and 2 jointly. (d) Extension vs. time plot displaying the 17 SMD trajectories that resulted in complete Csp unfolding. The dashed horizontal lines represent the mean length of the 5 intermediates and the fully unfolded state obtained from the statistical analysis of all the conducted SMD simulations.
